# A Novel Frameshift Mutation in *SLC20A2* in a Korean Patient with Primary Brain Calcification, Parkinsonism and Memory Impairment

**DOI:** 10.3390/biomedicines14030675

**Published:** 2026-03-16

**Authors:** Eva Bagyinszky, Minju Kim, Young Ho Park, Danyeong Kim, Seong Soo A. An, SangYun Kim

**Affiliations:** 1 Department of Industrial and Environmental Engineering, Graduate School of Environment, Gachon University, Seongnam 13120, Republic of Korea; eva85@gachon.ac.kr; 2 Department of Neurology, Chungbuk National University Hospital, Cheongju 28644, Republic of Korea; kmj682908@gmail.com; 3 Department of Neurology, Seoul National University College of Medicine & Clinical Neuroscience Center, Seoul National University Bundang Hospital, Seongnam 13620, Republic of Korea; kumimesy@gmail.com; 4 Department of Bionano Technology, Gachon Medical Research Institute, Gachon University, Seongnam 13120, Republic of Korea; dan627328@gmail.com; 5 Department of Neurology, Veterans Medical Research Institute, Veterans Health Service Medical Center, Gangdong-gu, Seoul 05368, Republic of Korea

**Keywords:** SLC20A2, calcifications, motor impairment, cognition, haploinsufficiency primary brain calcification

## Abstract

Objectives: The patient presented various neurological symptoms in her 50s, such as memory issues, insomnia, depression, and motor impairment. Diverse investigations were performed to identify the underlying causes on her neurological symptoms and understand her neuro- deteriorations. Methods: Clinical neurological and brain imaging analyses: CT, MRI and PET were performed on the patient. Blood was drawn for the whole-exome sequencing and functional studies with biomarker for amyloid-beta oligomers and SLC20A2 protein in plasma. Results: Brain imaging revealed calcifications in multiple regions, including the subcortical white matter, basal ganglia, thalami, and dentate nuclei. Genetic analysis revealed a c.1152_1153delCA, p.Asn384Lysfs*30 variant in *SLC20A2* gene. The decreased SLC20A2 protein levels in plasma in comparison to healthy controls suggested a loss-of-function mechanism from the mutation. The patient had a positive AlzOn result, indicating an increased tendency for amyloid oligomerization and suggesting a potential indirect link between SLC20A2 and amyloid-beta pathways. Conclusions: A novel frameshift mutation, Asn384Lysfs*30, in the *SLC20A2* gene was identified in a patient with Primary Brain Calcification (PBC). This mutation was located in a critical large loop region of the protein, where other similar mutations (e.g., Gly366fs89, Ser385Ilefs*70) were previously reported. These findings indicated that mutations in *SLC20A2* caused the reduced protein expressions, potentially resulting PBC through haploinsufficiency.

## 1. Introduction

Primary brain calcification (PBC) is a brain disease characterized by calcium deposits, called calcifications, in various brain regions. Calcifications primarily affect the basal ganglia, but they may occur in other areas, including the thalamus, subcortical white matter, or dentate nucleus. Disease age of onset may have a wide range, since it can occur in childhood, young adulthood or later adulthood. Clinical symptoms may be similar to other adult-onset neurodegenerative diseases, such as Alzheimer’s disease (AD), Parkinson’s disease (PD) or frontotemporal dementia (FTD). Diverse clinical phenotypes were reported in patients with PBC, including motor dysfunctions (such as clumsiness, muscle cramping or unstable gait), difficulties in concentration, memory impairments, personality changes, dementia, psychosis or headache [1,2,3,4,5].

Several genes were identified as causative factors for PBC, and they may act through loss-of-function mechanisms. Both autosomal dominant and recessive forms of PBC were reported. Autosomal dominant PBC was associated with mutations in the *SLC20A2*, *PDGFRB (platelet-derived growth factor receptor-β), PDGFB (platelet-derived growth factor subunit B)*, or *XPR1 (xenotropic and polytropic retrovirus receptor 1)* genes. Autosomal recessive PBC was linked to mutations in *MYORG (myogenesis regulating glycosidase), JAM2 (junctional-adhesion-molecule-2), CMPK2 (uridine monophosphate-cytidine monophosphate kinase 2) genes,* or *N-alpha-acetyltransferase 60 (NAA60)* [1,2,3,4,5,6]. Furthermore, potential candidates for PBC were also reported, including *Cytidine/Uridine Monophosphate Kinase 2 (CMPK2)* and *ribosomal RNA Processing Protein Coding (RRP12)* genes. Both genes were reported to be inherited in autosomal recessive form; however, their impact in PBC should be verified by independent replication studies [7,8].

*SLC20A2* gene, located on chromosome 8, encodes a type-III sodium-dependent phosphate transporter, PiT2, involved in phosphate homeostasis in neurons, astrocytes, and endothelial cells. Deficiency of SLC20A2 may result in motor, cognitive, and behavioral dysfunctions, and calcium deposits in the brain [1,2,3,4]. In this study, a frameshift mutation in *SLC20A2*, Asn384Lysfs*30, was found in a Korean patient with PFC, parkinsonism, memory dysfunctions, insomnia and depression. Reduced SLC20A2 levels were detected in plasma, suggesting loss-of-function mechanisms from the mutation.

## 2. Materials and Methods

### 2.1. Patient Information

**Figure 1:**
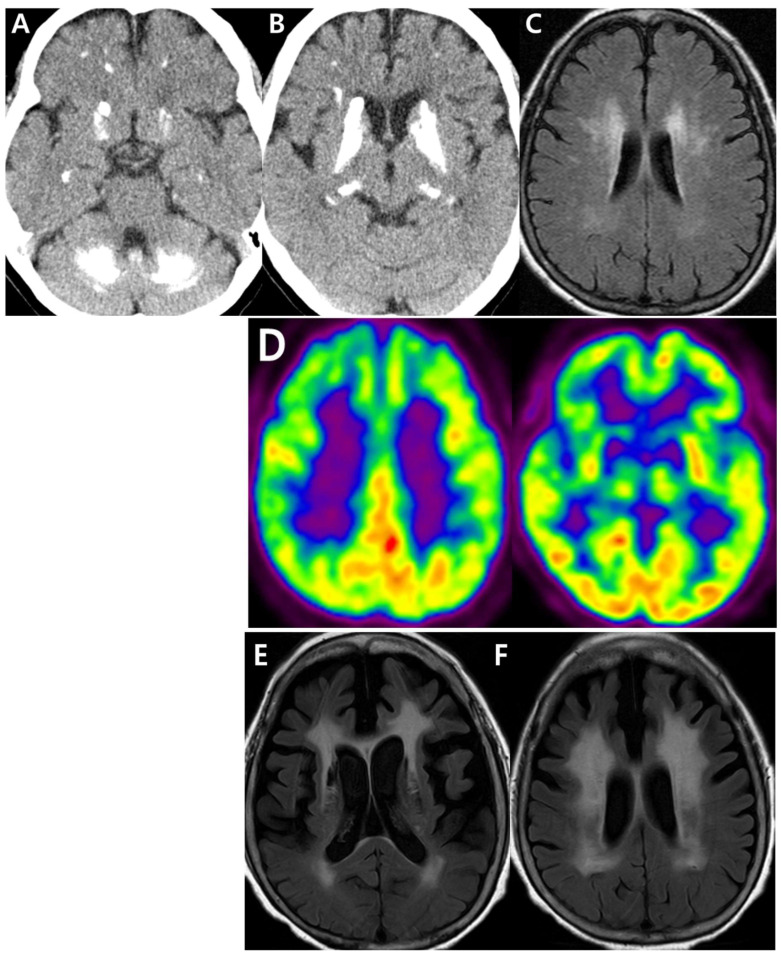
Imaging data of proband patient. (**A**,**B**) CT imaging of the patient at the age of 61 revealed calcification in the bilateral subcortical white matter, basal ganglia, thalami, and dentate nuclei. (**C**) FLAIR-MRI imaging at her initial visit showed severe diffuse periventricular white matter hyperintensities. (**D**) PET imaging showed hypometabolism in the frontal, temporal, parietal cortex, basal ganglia, and right thalamus. Red meant the high brain metabolism, green meant the moderate brain metabolism, while blue and purple meant the reduced brain metabolism (**E**,**F**). MRI FLAIR images obtained at the age of 77, with observed progression of periventricular white matter hyperintensities.

The 61-year-old female patient visited Seoul National University Bundang Hospital with a ten-year history of subjective memory problems, depressive mood and insomnia. Physical examination during her first visit revealed no motor impairments (e.g., rigidity, tremor, weakness). Brain computed tomography (CT) revealed calcifications in the bilateral subcortical white matter, basal ganglia, thalami, and dentate nuclei (Figure 1A,B). Magnetic resonance imaging (MRI) of the brain indicated severe diffuse periventricular white matter hyperintensities on Fluid-Attenuated Inversion Recovery (FLAIR) sequences (Figure 1C). Positron Emission Tomography with 18F-Fluorodeoxyglucose (F-18 FDG-PET) imaging showed hypometabolism in the bilateral frontal, temporal, and parietal cortices, basal ganglia, and right thalamus (Figure 1D). The total calcification score (TCS) was measured by the TCS tool, based on the full CT scan sequence (https://pbc-tcstool.github.io/ accessed on 2 March 2026). The TCS score was 43, which suggested a mild to moderate degree of calcification (Supplementary Table S1). Laboratory tests showed that serum calcium, phosphate, thyroid hormone, and parathyroid hormone levels were all normal.

Thirteen years later, the patient developed hand and foot tremors, decreased voice volume, and postural instability. The patient was treated with levodopa (100 mg) and benderizine (25 mg) under the suspicion of PD or atypical parkinsonism. No definite clinical improvement was observed. Follow-up brain MRI FLAIR images obtained during outpatient visits showed progression of periventricular white matter hyperintensities, which had become extensive (Figure 1E,F). The patient’s Clinical Dementia Rating score increased from 0.5 at her first visit to 1 at the most recent evaluation, with the sum of boxes score rising from 2.5 to 6 and the Mini-Mental State Examination score declining from 26 to 21. UPDRS part III was not performed, but it will be performed in a follow-up study.

Despite the absence of lacunar infarcts typically associated with cerebral small vessel disease (SVD) and the lack of vascular risk factors, the patient exhibited a progressively worsening clinical course, specifically indicating progression of SVD, accompanied by extensive and atypical calcifications. Consequently, whole-exome sequencing was conducted to investigate the possibility of underlying genetic mutations. There was no relative affected by similar symptoms; however, family members refused to go under genetic testing. This study was approved by the Institutional Review Board of Seoul National University Bundang Hospital (B-2508-988-701).

### 2.2. Methods

Total genomic DNA was extracted from white blood cells (WBCs) using the Qiagen blood kit (Seoul, Republic of Korea). Whole-exome sequencing was performed by Macrogen using the Illumina platform (https://www.macrogen.com, Seoul, Republic of Korea). The patient was screened for several genetic factors associated with neurodegenerative diseases, including Alzheimer’s disease (AD), frontotemporal dementia, motor diseases (PD, amyotrophic lateral sclerosis), and vascular diseases [9]. Whole-exome sequencing data was uploaded to the Franklin Genoox tool, which was an online platform designed for classifying genetic variants. This tool was used to analyze variants in multiple public and proprietary databases, including ClinVar, dbSNP, gnomAD, and HGMD, and provides comprehensive variant information (https://franklin.genoox.com/clinical-db/home, accessed on 1 March 2024) [6]. Candidate variants were confirmed by Sanger sequencing. Furthermore, functional verification of the *SLC20A2* frameshift mutation in plasma was performed in comparison to healthy controls using an ELISA kit (MyBiosource; MBS9338960, San Diego, CA, USA). The blood was tested for amyloid-beta (Aβ) oligomerization tendency using AlzOn (PeopleBio Inc., Sungnam, Republic of Korea).

## 3. Results

### 3.1. Genetic Analysis

**Figure 2:**
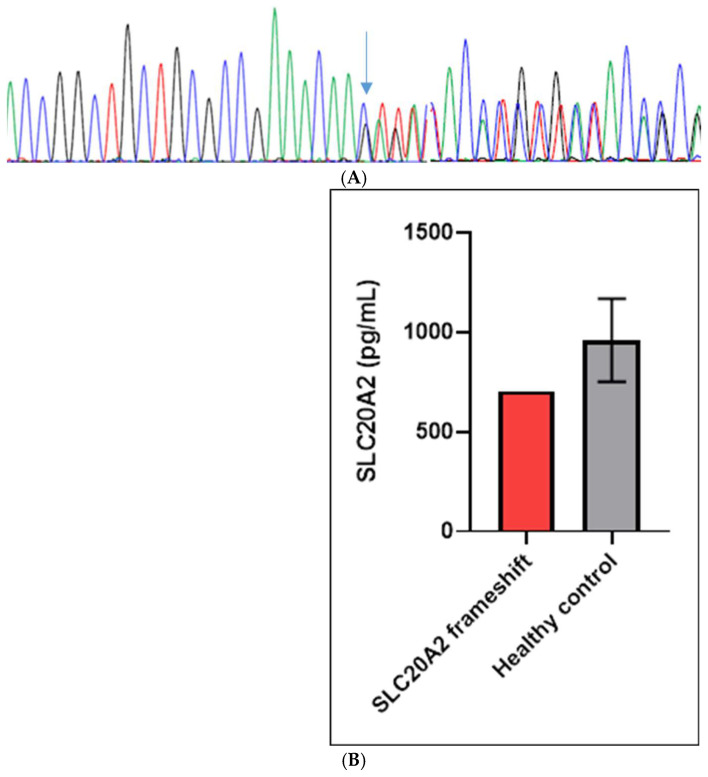
(**A**). Sanger sequencing data of patient with SLC20A2 Asn384Lysfs*30 frameshift variant The arrow shows the mutation site. (**B**). SLC20A2 levels in the proband patient and the healthy controls.

Franklin Genoox analysis revealed a rare frameshift mutation in the *SLC20A2* gene (g. 8:42437358:CTG>C; c.1152_1153delCA, p.Asn384Lysfs*30 variant, rs775204334). This mutation was predicted to result in a premature stop codon 30 residues downstream of the frameshift, and it was confirmed by Sanger sequencing (Figure 2A). *SLC20A2* Asn384Lysfs*30 was absent in the 1000Genomes database, but it was found in the GnomAD database (https://gnomad.broadinstitute.org) in one American and two non-Finnish European individuals; it was not observed in East Asians or South Asians. No additional pathogenic or likely pathogenic variants were found in other PBC-related genes or PBC candidates [1,2,3,4,5,6,7,8]. Furthermore, no pathogenic or likely pathogenic mutations were observed when analyzing the patient using our gene panel for several neurodegenerative diseases, including other neurodegenerative risk factors such as AD, FTD, or PD [9] (Supplementary Table S2).

### 3.2. Biomarker Studies

The concentrations of SLC20A2 protein in the plasma were lower in the patient compared to healthy controls (Figure 2B, Supplementary Table S3, Supplementary Figure S1). Furthermore, the patient showed an increased tendency for beta-amyloid oligomerization. The AlzOn assay yielded a positive result (value: 1.001), indicating an increased tendency for Aβ oligomerization.

## 4. Discussion

A frameshift mutation, Asn384Lysfs*30 in the *SLC20A2* gene, was discovered in a 61-year-old Korean patient with probable de novo PBC. CT analysis revealed calcifications in different brain areas, including the bilateral subcortical white matter, basal and ganglia. Her initial symptoms were insomnia and depression, followed by foot tremors and postural instability.

The levels of SLC20A2 protein were reduced in the patient compared to age-matched controls, suggesting the presence of a loss-of-function mechanism. Interestingly, AlzOn findings indicated positive results for amyloid oligomers. However, potential AD pathology was not proven because the cerebrospinal fluid (CSF) could not be obtained for measuring biomarkers, including the Ab42/Ab40 ratio, p-Tau, or t-Tau. Furthermore, amyloid PET imaging was not performed. The positive AlzOn results suggested a possible involvement of SLC20A2 in amyloid pathology. It may be possible that SLC20A2 dysfunction indirectly impacted the amyloid metabolism. One explanation could be that abnormal phosphate transport could disrupt calcium metabolism, leading to calcium deposition in the brain, leading to increased amyloid accumulation [10,11]. Frentz et al. (2024) [12] revealed that patients with arterial calcification and amyloid biomarkers presented worse cognition, suggesting that SLC20A2 may impact amyloid metabolism and cognitive decline in proband patients. However, additional investigations would be needed to clarify the relationship between SLC20A2 dysfunctions and amyloid metabolism [12].

**Figure 3:**
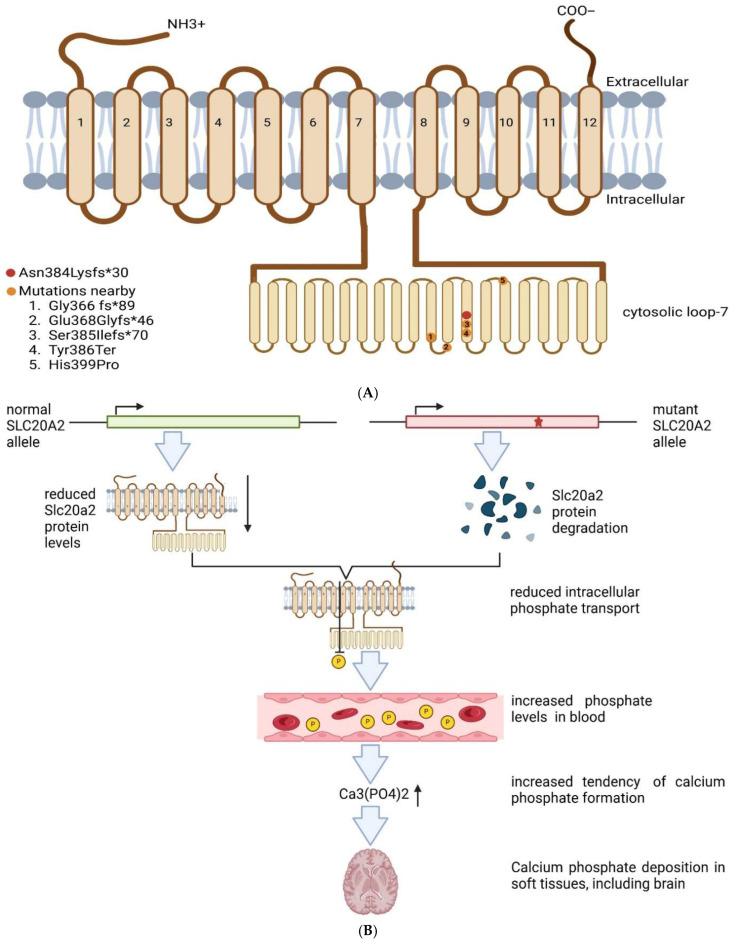
(**A**). Structure of SLC20A2 protein and location of mutations in the loop 7, including the Asn384Lysfs*30 mutation [13,14,15,16,17,18,19,20] (**B**). Loss-of-function mechanisms as a result of SLC20A2 haploinsufficiency. The arrow means reduced levels of SLC20A2 protein, and increased levels of calcium phosphate. Star means the frameshift mutation here.

The Asn384Lysfs*30 mutation was located in the large loop-7 of the SLC20A2 protein, which may impact PiT2 protein transport to the cell surface. This region was suggested to be involved in neuronal overgrowth and PiT2 interactions with microtubule-associated protein 1B (MAP1B). Abnormal SLC20A2 function was reported to reduce phosphate transport into cells and the elevate phosphate levels in the bloodstream, leading to risk of calcium phosphate formation and calcium deposition in the brain [13,14,15,16,17,18,19,20] (Figure 3A,B). Several mutations were reported near Asn384Lysfs*30 (Table 1). The Gly366fs mutation was found in a 57-year-old male patient, who developed motor and language impairment. CT revealed calcifications in several brain areas, e.g., the bilateral caudate nucleus, bilateral thalamus, bilateral cerebellum, and bilateral parietal lobes [15]. The Glu368Glyfs*46 mutation was identified in a familial case of PBC, where segregation was proven among affected relatives. The proband developed behavioral issues in her 40s, followed by motor and vocal dysfunctions in her 50s and cognitive decline in late disease stages. Calcifications were detected in her basal ganglia, thalamus, occipital cortex and dentate nuclei. The patient’s son was diagnosed with bipolar disorder and excessive eye blinking. His first CT showed calcifications in the basal ganglia and thalamic nuclei, which later spread to cerebellar dentate nuclei [16]. A patient with Ser385Ilefs*70 was found in sporadic PBC and presented motor impairments, including involuntary movement of the left limbs and bradykinesia in his 40s. Brain CT revealed calcification in multiple brain areas, including the caudate nuclei, lentiform nuclei, thalami, and cerebellar dentate nuclei [17]. The Tyr386Ter mutation was observed in a 33-year-old patient with migraine and mild functional impairment. CT revealed calcifications in different areas, including the globus pallidus and pulvinar region of the thalamus and dentate nucleus. RT-qPCR revealed a mild decrease (−10%) in SLC20A2 expression [18]. The His399Pro mutation was reported in a 41-year-old female patient with migraine and aura. CT showed calcifications in the bilateral putamen, and mild calcifications were found in the bilateral globus pallidus and dentate nuclei. CT performed 4 years later showed an approximately 30% elevation in calcification volume [19]. Table 1 compared the symptoms of mutations located near the novel *SLC20A2* Asn384fs*30 mutation.

In conclusion, a rare variant of Asn384Lysfs*30 in *SLC20A2* was identified in a Korean patient with PBC. Phenotypes of PBC with *SLC20A2* mutations may be similar to other neurodegenerative diseases, including AD, PD, depression or migraine [1,2,3,4,5,20,21]. Plasma analysis revealed reduced SLC20A2 protein levels from the plasma of the patient with *SLC20A2* Asn384Lysfs*30 mutation, leading to pathogenic pathways through haploinsufficiency. According to ACMG–AMP criteria [5], the *SLC20A2* c.1152_1153delCA (p.Asn384Lysfs*30) variant was classified as probably pathogenic, supporting a predicted loss-of-function effect, functional protein reduction, rarity in population databases, and a highly specific PBC phenotype. Interestingly, the co-existence of parkinsonism and a high tendency for amyloid oligomerization in the patient could explain the poor response to Levidopa and progressive white matter changes. These results suggested that further research may be needed to investigate the association between amyloid-related pathways and phosphate–calcium dysregulation, as well as the role of SLC20A2 in these pathways. In the future, cell studies with CRISPR-CAS9 should be performed on *SLC20A2* Asn384Lysfs*30 mutation to verify its loss-of-function mechanisms.

A limitations of this study was that we could not obtain enough WBCs from the patient for qPCR analysis. We also could not get obtain to confirm AD pathology from brain pathology, even though the amyloid oligomers were positive. Furthermore, all living family members refused the genetic test, hence the segregation analysis could not be carried out. Animal models were performed to examine the effects of antisense oligonucleotides (ASOs) in the case of SLC20A2 haploinsufficiency. These mouse models suggested that ASO administration resulted in elevated inorganic phosphate levels in CSF of mice, resulting in reduced levels of brain calcification. In the case of patients with *SLC20A2* haploinsufficiency, treatment in ASO may be promising, since it could slow down the disease progression [21,22,23,24]. Furthermore, astrocyte-mediated expression of SLC20A2 may also improve inorganic phosphate homeostasis inside the brains of *SLC20A2* knock-in mice, leading to a reduced degree of brain calcification [25].

## Figures and Tables

**Table 1:** Mutations in *SLC20A2* reported near Asn384Lysfs*30.

Mutation	Calcifications	Family History	Age of Onset	Symptoms	References
Gly366 fs*89	Bilateral caudate nucleus, bilateral thalamus, bilateral cerebellum, and bilateral parietal lobes	Probable positive	57	Motor and language impairment	[15]
Glu368Glyfs*46	Basal ganglia, left thalamic nucleus, dentate nuclei, occipital region	Probable positive	57	Depression, vocal and motor impairments, stroke	[16]
Asn384Lysfs*30	Subcortical white matter, basal ganglia, thalami, and dentate nuclei	Probable negative	Late 50s	Depression, insomnia, motor impairment	Our findings
Ser385Ilefs*70	Caudate nuclei, lentiform nuclei, thalami, and dentate nuclei of cerebellum	Probable positive	45	Bradykinesia	[17]
Tyr386Ter	Globus pallidus and pulvinar region of the thalamus, dentate nucleus, white matter of the cerebellum, and frontal subcortical regions	De novo	33	Migraine, mild functional impairment	[18]
His399Pro	Bilateral putamen, bilateral globus pallidus, and dentate nuclei	NA	37	Migraine without aura	[19]

## Data Availability

The data generated and analyzed during this study can be found within the manuscript and its Supplementary Files.

## Supplementary Materials

The following supporting information can be downloaded at https://www.mdpi.com/article/10.3390/biomedicines14030675/s1: Figure S1: Standard curve analysis of SLC20A2 ELISA; Table S1: Calcification scores, based on the brain CT series; Table S2: Variants, found by the gene panel for neurodegenerative diseases; Table S3: ELISA result of proband patient, compared to unaffected healthy controls.
