# Supplementary material for: A Novel Frameshift Mutation in SLC20A2 in a Korean Patient with Primary Brain Calcification, Parkinsonism and Memory Impairment
Source: Biomedicines. 2026 Mar 16;14(3):675. doi: 10.3390/biomedicines14030675 (PMC13023793; doi:10.3390/biomedicines14030675)
Supplement: Supplementary file 1 [file biomedicines-14-00675-s001.zip › biomedicines-4154469-supplementary.pdf]

Supplementary material

Supplementary Figure S1. Standard curve analysis of SLC20A2 ELISA

Supplementary figure S1.

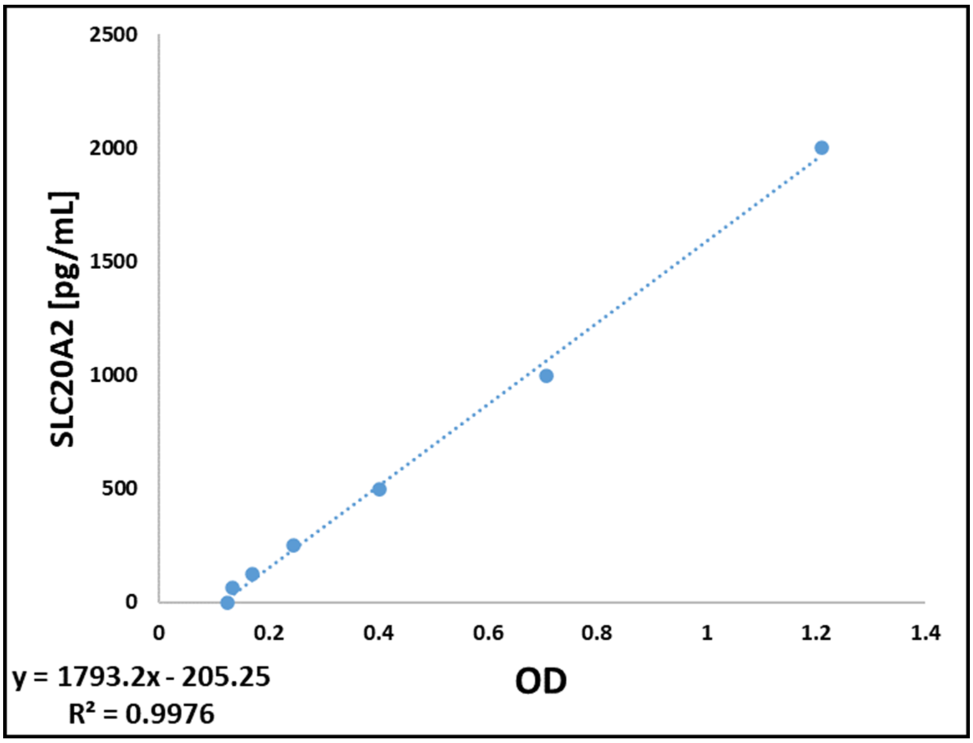

Supplementary tables legend

Supplementary Table S1: Calcification scores, based on the brain CT series

Supplementary Table S2. Variants, found by the gene panel for neurodegenerative diseases

Supplementary Table S1

Brain area	Calcification score
Left Lenticular Nucleus	4
Right Lenticular Nucleus	4
Left Caudate Nucleus	4
Right Caudate Nucleus	4
Left Thalamus	4
Right Thalamus	4
Left Cerebral Subcortical White Matter	2
Right Cerebral Subcortical White Matter	2
Cerebral Cortex	4
Left Cerebellar Hemisphere	4
Right Cerebellar Hemisphere	4
Vermis	3
Left Midbrain	0
Right Midbrain	0
Pons	0
Medulla	0
**Total Calcification Score (TCS)**	**43**

Supplementary Table S2

Gene	mRNA	protein	rsID	gnomAD_exomes	gnomAD_exomes_EAS_AF	SIFT_score
ABCA13	c.1517C>T	p.Pro506Leu	rs1880738	0.449307	0.575825	0.436,T
	c.6533C>A	p.Ala2178Glu	rs1880736	0.723538	0.989164	1,T
	c.8020C>T	p.Arg2674Trp	rs2222648	0.803456	0.991853	1,T
	c.8797G>A	p.Val2933Met	rs79809732	0.010481	0.080848	0.118,T
	c.9425C>T	p.Ala3142Val	rs3931814	0.133307	0.104824	0.918,T
	c.12829A>G	p.Asn4277Asp	rs4917152	0.172627	0.239183	1,T
	c.12905A>G	p.Gln4302Arg	rs4917153	0.172591	0.239214	0.64,T
ABCA7	c.563A>G	p.Glu188Gly	rs3764645	0.497496	0.428977	0.647,T
	c.4046G>A	p.Arg1349Gln	rs3745842	0.427897	0.335604	0.546,T
	c.4580G>C	p.Gly1527Ala	rs3752246	0.840229	0.646508	0.877,T
	c.6133G>T	p.Ala2045Ser	rs4147934	0.71193	0.446435	0.962,T
ACOX1	c.936C>G	p.Ile312Met	rs1135640	0.653791	0.800441	0.256,T
	c.301G>A	p.Gly101Ser	rs3744032	0.054032	0.132726	0.143,T
ALS2	c.1102G>A	p.Val368Met	rs3219156	0.912953	0.999826	0.191,T
ARAP2	c.4568G>A	p.Arg1523Gln	rs4833069	0.992128	1	1,T
ATP13A2	c.3214G>A	p.Ala1072Thr	rs3170740	0.47141	0.295664	0.988,T
ATXN1	c.1481T>C	p.Val494Ala	rs1164069483	NA	NA	0.002,D
	c.672_677delGCAGCA	p.Gln224_Gln225del	rs754954093	NA	NA	NA

	c.639G>T	p.Gln213His	rs3817753	NA	NA	0.072, T
	c.633T>G	p.His211Gln	rs59310777	NA	NA	0.297, T
	c.627_629delTCA	p.His209del	rs751377396	NA	NA	NA
	c.627T>G	p.His209Gln	rs11969612	NA	NA	0.065, T
ATXN1	c.624G>T	p.Gln208His	rs28555263	NA	NA	1,T
ATXN2	c.563_565delAGC	p.Gln188del	rs10560189;rs67658094	NA	NA	NA
	c.319C>G	p.Leu107Val	rs695871	0.768519	0	0.315, T
BST1	c.434G>A	p.Arg145Gln	rs2302464	0.047079	0.208164	0.111, T
CASS4	c.503C>T	p.Thr168Ile	rs77627768	0.002568	0.022556	0.024, D
	c.1978C>T	p.Pro660Ser	rs35031530	0.048838	0.254697	0.286, T
CD33	c.41C>T	p.Ala14Val	rs12459419	0.308116	0.184375	0.029, D
CDH12	c.100G>C	p.Glu34Gln	rs117878510	0.001683	0.022097	0.38,T
CIART	c.569C>T	p.Thr190Ile	rs116854758	0.00201	0.028235	0.35,T
COL4A1	c.4002A>C	p.Gln1334His	rs3742207	0.304165	0.251565	0.122, T
	c.19G>C	p.Val7Leu	rs9515185	0.435687	0.633385	0.575, T
CR1	c.4973A>G	p.His1658Arg	rs2274567	0.250551	0.291633	0.897, T
	c.5573C>T	p.Thr1858Met	rs3737002	0.282939	0.331399	0.019, D
	c.6178A>T	p.Thr2060Ser	rs4844609	0.985426	1	0.804, T
	c.6193A>G	p.Ile2065Val	rs6691117	0.321622	0.3062	1,T

	c.6830C>G	p.Pro2277Arg	rs3811381	0.241761	0.288787	0.446, T
	c.7255A>G	p.Thr2419Ala	rs2296160	0.819882	0.655775	0.987, T
CTNNA3	c.1787G>A	p.Ser596Asn	rs4548513	0.40376	0.431517	1,T
CTSA	c.108_110delGCT	p.Leu37del	rs1457838268	NA	NA	NA
DSG2	c.1984G>A	p.Ala662Thr	rs1186896680	4.07E-06	0	0,D
DSG2	c.2318G>A	p.Arg773Lys	rs2278792	0.265563	0.466526	0.383, T
EPHA1	c.2698A>G	p.Met900Val	rs6967117	0.940039	0.998493	1,T
	c.479T>C	p.Val160Ala	rs4725617	0.93221	0.991984	0.246, T
FNDC1	c.106T>C	p.Ser36Pro	rs295332	0.540541	0	0.981
	c.1312A>G	p.Thr438Ala	rs509648	0.324572	0.751479	1,T
	c.1387G>C	p.Glu463Gln	rs420137	0.866743	0.637705	1,T
	c.3007C>G	p.Gln1003Glu	rs370434	0.867677	0.641117	1,T
	c.3540C>G	p.Asp1180Glu	rs420054	0.850326	0.622257	1,T
	c.3782T>C	p.Leu1261Pro	rs3003174	0.86882	0.641139	0.345, T
	c.3839A>G	p.Gln1280Arg	rs2501176	0.869494	0.643025	1,T
	c.4436_4453delCCCGCCGCACGACCACCA	p.Thr1479_Thr1484del	rs141435210;rs3842694	NA	NA	NA
	c.4511C>A	p.Thr1504Lys	rs386360	0.872142	0.644028	0.795, T
	c.4720A>G	p.Thr1574Ala	rs7763726	0.064727	0.318933	0.042, D
FOXC1	c.1139_1141dupGCG	p.Gly380dup	rs545470261;rs76840944	NA	NA	NA
	c.1359_1361dupCGG	p.Gly454dup	rs572346201	NA	NA	NA
	c.121_123dupGCC	p.Ala41dup	rs752348150	NA	NA	NA

FOXF2	c.917_919dupGCG	p.Gly306dup	rs147426137;rs397731476;rs58230522	NA	NA	NA
GIGYF2	c.3693_3695delACA	p.Gln1232del	rs10555297	NA	NA	NA
GPNMB	c.881C>T	p.Ser294Phe	rs35499907	0.012057	0.125116	0.002, D
HIP1R	c.1873C>T	p.Arg625Trp	rs117866676	0.00527	0.066624	0.036, D
LAMP3	c.952A>G	p.Ile318Val	rs482912	0.651055	0.506262	1,T
LPA	c.6046C>T	p.Arg2016Cys	rs3124784	0.237652	0.125406	0.039, D
	c.5036T>C	p.Met1679Thr	rs1801693	0.642298	0.451334	1,T
	c.3476C>T	p.Thr1159Met	rs187364344	0.000313	0.002325	0.012, D
LRRK2	c.149G>A	p.Arg50His	rs2256408	0.993066	1	1,T
	c.4939T>A	p.Ser1647Thr	rs11564148	0.298352	0.33871	0.953, T
	c.7190T>C	p.Met2397Thr	rs3761863	0.618476	0.46794	0.466, T
MAPT	c.1321T>C	p.Tyr441His	rs2258689	0.281649	0.632031	0.978, T
MS4A4A	c.154A>G	p.Lys52Glu	rs10750931	0.137984	0.116187	0.077, T
	c.532A>G	p.Met178Val	rs6591561	0.292525	0.376595	0.837, T
MS4A6E	c.16A>G	p.Ile6Val	rs2304935	0.325262	0.270819	1,T
	c.28A>G	p.Thr10Ala	rs2304934	0.325243	0.27093	0.086, T
	c.139G>T	p.Val47Phe	rs2304933	0.325162	0.270275	0.044, D
NEK1	c.2255A>G	p.Glu752Gly	rs34099167	0.098048	0.167733	0.088, T
NME8	c.622T>C	p.Cys208Arg	rs10250905	0.731553	0.560475	0.046, D

NOTCH4	c.349A>C	p.Lys117Gln	rs915894	0.351497	0.46229	0.399, T
	c.45_47delGCT	p.Leu16del	rs35795312	NA	NA	NA
PDIA4	c.160G>C	p.Asp54His	rs144700814	0.000566	0.007964	0.004, D
PDLIM5	c.41C>T	p.Ser14Phe	rs2452600	0.265912	0.374971	0.024, D
	c.1120G>A	p.Ala374Thr	rs966845	0.993729	1	0.709, T
	c.1228A>G	p.Thr410Ala	rs7690296	0.44145	0.399779	0.433, T
	c.1562G>A	p.Ser521Asn	rs13107595	0.989681	1	0.74,T
PKP1	c.1244G>A	p.Gly415Asp	rs1626370	0.193315	0.232077	0.062, T
RIN3	c.2913_2915delCGG	p.Gly972del	rs570458246	NA	NA	NA
SACS	c.11032C>G	p.Pro3678Ala	rs17078601	0.040604	0.104027	0.052, T
	c.696T>A	p.Asn232Lys	rs2031640	0.112219	0.114649	0.214, T
SETX	c.7834A>G	p.Ser2612Gly	rs3739927	0.084295	0.387787	0.652, T
	c.7759A>G	p.Ile2587Val	rs1056899	0.38054	0.728828	1,T
	c.5563A>G	p.Thr1855Ala	rs2296871	0.263905	0.696578	0.83,T
SETX	c.1979C>G	p.Ala660Gly	rs882709	0.115986	0.44578	0.008, D
SFRP4	c.1019G>A	p.Arg340Lys	rs1802074	0.197331	0.243691	0.859, T
	c.958C>A	p.Pro320Thr	rs1802073	0.435974	0.550848	0.171, T
SIGMAR1	c.622C>T	p.Arg208Trp	rs11559048	0.007738	0.031196	0.011, D
SLC24A4	c.1654A>C	p.Lys552Gln	rs45587635	0.059926	0.228744	0.184, T
	c.485C>G	p.Ala162Gly	rs1443549	0.998607	1	1,T

SLC6A5	c.1371G>C	p.Lys457Asn	rs3740870	0.133103	0.067776	0.021, D
	c.1387G>A	p.Asp463Asn	rs1805091	0.225614	0.074684	0.399, T
SMC5	c.916G>A	p.Val306Ile	rs1180116	0.88404	0.855722	0.655, T
SORL1	c.1582G>A	p.Ala528Thr	rs2298813	0.072213	0.122273	0.306, T
	c.3220C>G	p.Gln1074Glu	rs1699107	0.996113	1	0.168, T
	c.5899G>A	p.Val1967Ile	rs1792120	0.996169	1	1,T
SPG11	c.1348A>G	p.Ile450Val	rs3759873	0.018511	0.096649	0.744, T
	c.833A>G	p.Asn278Ser	rs75309308	0.008068	0.102586	0.487, T
SYT11	c.144G>C	p.Gln48His	rs822522	0.990874	0.999942	0.866, T
TET1	c.485A>G	p.Asp162Gly	rs10823229	0.330584	0.383148	0.019, D
	c.577T>A	p.Ser193Thr	rs12773594	0.182135	0.172254	0.055, T
	c.767C>T	p.Ala256Val	rs12221107	0.115874	0.151786	0.094, T
	c.3053A>G	p.Asn1018Ser	rs16925541	0.108049	0.116531	0.09,T
	c.3369A>G	p.Ile1123Met	rs3998860	0.777419	0.839626	0.157, T
	c.6086C>A	p.Ala2029Asp	NA	NA	NA	0,D
TM2D3	c.17T>G	p.Leu6Arg	rs2939587	0.97935	1	0.311, T
TMEM106B	c.554C>G	p.Thr185Ser	rs3173615	0.489626	0.657474	0.214, T
TWF1	c.1060G>C	p.Ala354Pro	.	.	.	0.039, D
VEPH1	c.1564T>C	p.Ser522Pro	rs11918974	0.276265	0.392621	1,T

WDR64	c.3197T>C	p.Val1066Ala	rs151295225	0.00703	0.069414	0.024, D
ZCCHC14	c.160A>G	p.Ile54Val	rs11648852	0.14169	0.255943	0.949, D

Supplementary Table S3.

ID	MMSE scores	MRI Scan	PET Scan	Condition	SLC20A2 plasma concentration
**Control 1**	23	NA	Negative	Normal for her age	965.7096
**Control 2**	30	Mild SVD	Negative	Normal for her age	857.221
**Control 3**	26	Mild SVD	Negative	Normal for her age	742.4562
**Control 4**	30	Minimal SVD	ND	Normal for her age	987.228
**Control 5**	26	Minimal SVD	Negative	Normal for her age	1441.804
**Control 6**	28	Mild SVD<br>No change since 2021	ND	Normal for her age	883.2224
**Control 7**	28	Mild SVD	ND	Normal for her age	894.8782
**Control 8**	27	Minimal SVD	ND	Normal for her age	899.3612
**proband patient**	**21**	**extensive WMH**	**hypometabolism in several brain areas**	**motor impairment, memory dysfunctions**	**704.799**
